# Extended doxycycline treatment versus salpingectomy in the management of patients with hydrosalpinx undergoing IVF-ET

**DOI:** 10.1186/s13048-020-00665-0

**Published:** 2020-06-12

**Authors:** Usama M. Fouda, Hesham S. Elshaer, Mohamed A. Youssef, Fatma F. Darweesh

**Affiliations:** 1 Department of Obstetrics and Gynecology, Faculty of Medicine, Cairo University, Kasr Al-Ainy Hospital, Al-Saraya Street, Cairo, Egypt; 2 Riyadh Fertility and Reproductive Health Center, Giza, Egypt

**Keywords:** Salpingectomy, Doxycycline, Hydrosalpinx, IVF-ET, Infertility

## Abstract

**Background:**

The aim of this study was to determine whether the treatment with doxycycline before and after oocyte retrieval is as effective as salpingectomy in minimizing the detrimental effect of hydrosalpinx on the outcomes of IVF-ET.

**Methods:**

A retrospective analysis was done for the outcomes of the IVF-ET cycles of patients with hydrosalpinx who underwent laparoscopic salpingectomy prior to IVF cycle (*n* = 260) or were treated with extended doxycycline treatment during the IVF cycle (*n* = 45). In doxycycline group, doxycycline (100 mg twice daily) was started 1 week before anticipated oocyte retrieval and was continued for 1 week after oocyte retrieval. In salpingectomy group, the mesosalpinx was coagulated as close as possible to the fallopian tube.

**Results:**

The implantation, clinical pregnancy, ongoing pregnancy and live birth rates were significantly higher in the salpingectomy group (20.87% Vs. 9.91%, *P* value =0.007, 44.62% Vs. 20%, *P* value = 0.002, 39.62% Vs. 17.78%, *P* value = 0.005 and 37.31% Vs. 15.56%, *P* value = 0.005 respectively).

**Conclusion:**

Salpingectomy is more effective than extended doxycycline treatment in improving the outcomes of IVF-ET in patients with hydrosalpinx undergoing IVF-ET. Further, larger well designed randomized controlled trials should be conducted to confirm the findings of this study.

## Introduction

The negative consequences of hydrosalpinx on the outcomes of IVF-ET are confirmed by an overwhelming scientific evidence [1, 2]. A meta-analyses including 5592 patients with tubal factor of infertility (1004 with hydrosalpinx and 4588 without hydrosalpinx) revealed that the implantation, pregnancy and delivery rates were significantly lower in hydrosalpinx group (8.5% Vs. 13.7%, 19.7% Vs. 31.2%, 13.4% Vs. 23.4% respectively). Moreover, the miscarriage rate was significantly higher in hydrosalpinx group (43.7% Vs. 31.1%) [3].

Since 1994, there is almost agreement between studies that salpingectomy or proximal tubal occlusion significantly improve the reproductive outcomes of IVF-ET [1, 4, 5]. A Cochrane review revealed that salpingectomy or proximal tubal occlusion prior to IVF-ET significantly increased the clinical pregnancy, ongoing pregnancy and live birth rates [6]. However, salpingectomy or proximal tubal occlusion are invasive procedures that may be associated with peri-operative complications especially in patients with dense pelvic adhesions. Moreover, some patients decline to undergo bilateral salpingectomy or tubal occlusion because these procedures remove any possibility of spontaneous pregnancy.

Other less invasive and safer treatment modalities for patients with hydrosalpinx undergoing IVF-ET include ultrasound guided aspiration of hydrosalpinx fluid and hysteroscopic occlusion of fallopian tube with Essure micro-inserts. Although the initial results of these treatment modalities are promising, their routine use in the management of patients with hydrosalpinx undergoing IVF-ET is not recommended because their supporting evidence was obtained from small studies [7–9]. Moreover, ultrasound guided aspiration of hydrosalpingeal fluid may cause flaring of pelvic infection or injury of bowel and cannot be used in patients with hydrosalpinges which are not visible by ultrasound. Similarly, hysteroscopic occlusion of fallopian tube with Essure micro-inserts delays IVF cycle, may cause injury of uterus and may be associated with long term complications such as menstrual irregularities, allergy and chronic pelvic pain [7–9].

A small retrospective study has revealed that the reproductive outcomes of IVF-ET cycles were comparable in patients with hydrosalpinx treated with doxycycline (100 mg twice daily 1 week before expected oocyte retrieval and continued until 6 days after oocyte retrieval) versus patients with tubal occlusion/adhesion and patients with endometriosis/unexplained infertility [10].

The aim of this study was to determine whether the treatment with doxycycline before and after oocyte retrieval is as effective as salpingectomy in minimizing the detrimental effect of hydrosalpinx on the outcomes of IVF-ET.

## Materials and methods

We conducted a retrospective analysis for the outcomes of the IVF-ET cycles of patients with hydrosalpinx who underwent salpingectomy prior to the IVF cycle (*n* = 260) or were treated with extended doxycycline treatment during the IVF cycle (*n* = 45) in Riyadh fertility and reproductive health center during the period between 2012 and 2017. Laparoscopic salpingectomy was offered to all the patients with hydrosalpinx diagnosed with hysterosalpingography or laparoscopy. Patients who declined surgery or have extensive pelvic adhesions or have history of multiple laparotomies underwent ultrasound guided aspiration of hydrosalpingeal fluid or received doxycycline treatment.

In doxycycline group, doxycycline (100 mg twice daily) was started 7 days before anticipated oocyte retrieval and was continued for 7 days after oocyte retrieval. In laparoscopic salpingectomy group, the mesosalpinx was coagulated as near as possible to the fallopian tube using bipolar diathermy. IVF-ET cycles were started at least 2 months after salpingectomy. The first attempt fresh IVF-ET cycles after salpingectomy were included in the analysis.

Patients with age more than 37 years, anti-Müllerian hormone (AMH) ≤ 0.3 ng/ml, antral follicle count (AFC) < 6, uterine fibroid requiring surgical removal, endometriosis and history of recurrent miscarriage, were excluded from the study. Patients were informed about the possibility of using their medical records in research and they did not refuse. Ethics committee approved the protocol of the study. The patients were contacted to obtain their consent for the retrospective study.

A long GnRH agonist protocol using triptorelin (Triptofem, Karmed HandelsgesmbH, Austria) at a daily dose of 1 mg s.c was started 7 days before the anticipated date of the menstruation. Once pituitary down-regulation was achieved (confirmed by the absence of cysts in ovary, endometrial thickness less than 5 mm, serum estradiol less than 50 pg/ml), the dose of triptorelin was reduced to 0.5 mg and ovarian stimulation with highly purified urinary FSH (HP-uFSH) (Fostimon, IBSA, Switzerland) was started on cycle day 2 or 3. The starting dose of HP-uFSH varied from 150 IU/day to 300 IU/day depending on the age of the patient, AMH level, basal FSH level and antral follicle count. After the fifth stimulation day, the dose of HP-uFSH was adjusted according to the ovarian response. Human chorionic gonadotropin (HCG) (Pregnyl; N.V.Organon, Oss, Holland) at dose of 10,000 IU was administered intramuscularly when there were at least 3 follicles ≥17 mm in mean diameter. Ovum pickup was carried out by ultrasound-guided follicular fluid aspiration 36 ± 2 h after HCG administration.

A maximum of 3 embryos were transferred 3 to 5 days after ovum pickup. Pregnancy test was performed 2 weeks after embryo transfer and ultrasound examination was performed 5 weeks after embryo transfer to determine the number of gestational sacs inside uterus and to detect the presence of fetal cardiac activity [11]. Progesterone vaginal suppository (Prontogest, Marcyrl, Egypt) was administered at a daily dose of 800 mg for luteal phase support.

### Statistical analysis

Student t test was used to compare quantitative variables and Chi-square (χ2) test was used to compare categorical data. Yates correction equation was used instead of Chi-square (χ2) test when the expected frequency was less than 5. A probability value (*p* value) < 0.05 was considered statistically significant. Statistical calculations were performed using Microsoft Excel version 7 (Microsoft Corporation, NY).

## Results

There were no significant differences between both groups with respect to age, body mass index, basal FSH, type and duration of infertility, percentage of patients with bilateral hydrosalpinx and percentage of patients with ultrasound visible hydrosalpinx (Table 1).

**Table 1:** Patients^,^ characteristics

	Salpingectomy group (***n*** = 260)	Doxycycline group (***n*** = 45)	***P*** value
**Age (years)**	**27.65 ± 3.55**	**28.31 ± 3.71**	**0.274**
**Body mass index (Kg/m**^**2**^**)**	**26.17 ± 3.018**	**26.44 ± 3.35**	**0.608**
**Duration of infertility (years)**	**3.54 ± 1.82**	**3.27 ± 1.72**	**0.337**
**Type of infertility**			
**• Primary**	**212/260 (81.54%)**	**33/45 (73.33%)**	**0.201**
**• Secondary**	**48/260 (18.46%)**	**12/45 (26.67%)**	**0.201**
**Basal FSH (IU/L)**	**6.3 ± 2.01**	**6.19 ± 2.07**	**0.749**
**Bilateral hydrosalpinx**	**52/260 (20%)**	**10/45 (22.22%)**	**0.732**
**Hydrosalpinx visible by U/S**	**121/260 (46.54%)**	**23/45 (51.11%)**	**0.571**

Values are expressed as mean ± SD or n/n(%)

*U/S* Ultrasound

Table 2 shows the cycle characteristics in both groups. The stimulation period, total dose of HP-uFSH, number of follicles ≥17 mm on the day of HCG administration, oocytes retrieved, metaphase II oocyte, 2 pro-nucleate (2PN) embryos and number of embryos transferred were comparable between both groups.

**Table 2:** IVF cycle characteristics

	Salpingectomy group (***n*** = 260)	Doxycycline group (***n*** = 45)	***P*** value
**Stimulation period (days)**	**11.16 ± 3.52**	**11.53 ± 1.71**	**0.42**
**Consumed HP-uFSH units**	**2596 ± 502**	**2708 ± 650**	**0.278**
**Follicles ≥ 17 mm on the day of HCG administration**	**11.16 ± 3.52**	**11.89 ± 4.68**	**0.324**
**Retrieved oocytes**	**10.13 ± 3.2**	**10.64 ± 4.69**	**0.479**
**Metaphase II oocytes**	**8.7 ± 3.25**	**8.53 ± 3.23**	**0.76**
**Two pronucleate embryos**	**6.70 ± 2.63**	**6.96 ± 3.91**	**0.68**
**Fertilization rate**	**1609/2087 (77.1%)**	**313/384(81.51%)**	**0.056**
**No. of embryos transferred**	**2.59 ± 0.49**	**2.47 ± 0.55**	**0.146**
**Grade I & II embryos /transferred embryos**	**422/623 (67.74%)**	**72/111 (64.86%)**	**0.552**

Values are expressed as mean ± SD or n/n (%)

The implantation, clinical pregnancy, ongoing pregnancy and live birth rates were significantly higher in the salpingectomy group (20.87% Vs. 9.91%, *P* value =0.007, 44.62% Vs.20%, *P* value = 0.002, 39.62% Vs. 17.78%, *P* value = 0.005 and 37.31%Vs. 15.56%, *P* value = 0.005 respectively). The abortion rate was comparable between both groups (11.21% Vs. 11.11%, *P* value =0.993) (Table 3). Seven patients in doxycycline group have uterine fluid collection on the day of embryo transfer. No pregnancies occurred in those patients. None of the patients in salpingectomy group had uterine fluid collection on the day of embryo transfer.

**Table 3:** Reproductive outcomes

	Salpingectomy group (***n*** = 260)	Doxycycline group (***n*** = 45)	***P*** value
**Clinical pregnancy rate**	**116/260 (44.62%)**	**9/45 (20%)**	**0.002**
**Ongoing pregnancy rate**	**103/260 (39.62%)**	**8/45 (17.78%)**	**0.005**
**Live birth rate**	**97/260 (37.31%)**	**7/45 (15.56%)**	**0.005**
**Implantation rate**	**130/623 (20.87%)**	**11/111 (9.91%)**	**0.007**
**Spontaneous abortion rate n/IUP**	**13/116(11.21%)**	**1/9 (11.11%)**	**0.993**

Values are expressed as n/n (%)

*IUP* Intrauterine pregnancy

None of the patients in salpingectomy group had peri-operative complications and none of the patients in the doxycycline group had adverse drug effects.

## Discussion

The data presented in the current study revealed that salpingectomy is more effective than extended doxycycline treatment during IVF-ET cycle in improving the outcomes of IVF-ET in patients with hydrosalpinx undergoing IVF-ET.

The results of the current study indicate that the extended doxycycline treatment is not effective in minimizing the detrimental effect of hydrosalpinx on the outcomes of IVF-ET. In contrast to our findings, a retrospective study comparing the reproductive outcomes of IVF-ET cycles of 17 patients with hydrosalpinx treated with extended doxycycline treatment with the reproductive outcomes of IVF-ET cycles of 25 patients with adhesions/proximal tubal occlusion, and 22 patients with endometriosis/unexplained infertility revealed that the implantation and live birth rates were comparable between the three groups. The authors suggested that antibiotic treatment could prevent the detrimental impact of hydrosalpinx on the outcomes of IVF-ET [10].

In the current study, no pregnancies occurred in 7 patients in the doxycycline group who have uterine fluid collection on the day embryo transfer. The results of our study are in agreement with several studies which revealed that the patients with hydrosalpinx and uterine fluid collection on the day embryo transfer have almost no chance of pregnancy if embryos are transferred [12–14].

Hydrosalpinx is a chronic pathological condition characterized by accumulation of clear watery fluid in the fallopian tube due to obstruction of the distal end of the fallopian tube. Obstruction of the distal end of fallopian tube usually occur as a result of acute salpingitis. Other rare causes of obstruction of distal end of fallopian tube include endometriosis, appendicitis or pelvic adhesions [1, 2].

Several theories have been proposed to explain the association between hydrosalpinx and poor outcomes of IVF-ET. Several authors proposed that the leakage of hydrosalpingeal fluid into the uterine cavity may exert an embryotoxic effect, alter endometrial receptivity or mechanically wash the embryos [15–17].

Although the majority of studies documented the toxicity of hydrosalpingeal fluid on mouse embryos [15], several studies revealed that the hydrosalpingeal fluid has no adverse effect on human embryos development [16, 17].

One theory suggested that the inflammation of the hydrosalpinx (caused by Chlamydia trachomatis or other bacteria) is the main cause of the detrimental effect of hydrosalpinx on the outcomes of IVF-ET. Consequently, antibiotics (particularly those effective against Chlamydia trachomatis) were used to prevent this detrimental effect [10].

Several authors suggested that cytokines, prostaglandins, leukotrienes and reactive oxygen species produced by chronic inflammatory cells associated with hydrosalpinx could have an embryotoxic effect [18, 19]. Moreover, simultaneous acute salpingitis and acute endometritis often occur in patients with acute pelvic inflammatory disease (PID), puerperal sepsis or post abortion infections. Hydrosalpinx and chronic endometritis are common sequels of acute salpingitis and acute endometritis respectively [20]. Several studies revealed that chronic endometritis can cause recurrent implantation failure and that antibiotic treatment of chronic endometritis could improve the uterine receptivity [21, 22].

Several studies revealed that the expression of markers of endometrial receptivity (alpha v  beta 3 and leukaemia inhibitory factor) was significantly decreased at the time of the implantation window in patients with hydrosalpinx and that the expression of these markers was significantly increased after salpingectomy [23, 24]. The results of these studies explained the mechanism by which hydrosalpinx exerts its detrimental effect on the outcomes of IVF-ET and confirmed that salpingectomy can prevent this effect.

The main limitations of the current study are the retrospective design and the small sample size. However, to the best of our knowledge the current study is the largest study which evaluated the effect of extended doxycycline treatment before and after oocyte retrieval on the outcomes of IVF-ET cycles in patients with hydrosalpinx. Moreover, the current study is the first study which compared salpingectomy with extended doxycycline treatment before and after oocyte retrieval in the management of patients with hydrosalpinx undergoing IVF-ET.

## Conclusion

In conclusion, salpingectomy is more effective than extended doxycycline treatment in improving the outcomes of IVF-ET in patients with hydrosalpinx undergoing IVF-ET. Further, larger well designed randomized controlled trials should be conducted to confirm the findings of this study.

## Data Availability

The datasets used and/or analysed during the current study are available from the corresponding author on reasonable request.

## Abbreviations

AFC: Antral follicle count
AMH: Anti-Müllerian hormone
FSH: Follicle stimulating hormone
ICSI: Intra cytoplasmatic sperm injection
IVF-ET: In vitro fertilization embryo transfer
IUP: Intrauterine pregnancy
PID: Pelvic inflammatory disease
U/S: Ultrasound
